# An interventricular membranous septal aneurysm obstructing the right ventricle outflow tract in a five-year-old boy: a case report

**DOI:** 10.1186/s13019-020-01349-y

**Published:** 2020-10-02

**Authors:** Lubna Bakr, Mohammad Al-Jadaan, Mohammad Younes

**Affiliations:** Department of Cardiac Surgery, Faculty of Medicine, Damascus University, Damascus, Syria

**Keywords:** Aneurysm of the membranous septum, Right ventricular outflow tract, Ventricular septal defect, Cardiac surgery, Case report

## Abstract

**Background:**

While the aneurysms of the membranous septum (AVS) are rare, the possibility that they lead to obstruction is even rarer. To the best of our knowledge, 11 similar cases have been reported since 1982.

**Case presentation:**

Initially, the five-year-old boy was evaluated for dyspnoea that had been present since birth. He did not receive any medical treatment until the previous year. At the age of four, the transthoracic echocardiography showed a large aneurysm extending to the right ventricular outflow tract (RVOT) and causing RVOT stenosis. Complete surgical resection of the aneurysmal tissue was performed, and the boy was discharged home in satisfactory condition.

**Conclusions:**

As the occurrence of RVOT obstruction by a membranous ventricular septal aneurysm is very rare, we are reporting the second case in which an aneurysm of the membranous septum dynamically obstructed the RVOT in a child. We are also reviewing all the previously reported similar cases in the literature. Further studies are needed to obtain a more comprehensive understanding of aneurysms of the membranous septum (AVS).

## Background

An aneurysm of the membranous portion of the ventricular septum is a rare congenital anomaly, occurring alone or in combination with other cardiac lesions, and is usually of no clinical significance [1]. Ventricular septal defect (VSD) is the most common congenital heart malformation (30–40%), as an isolated finding [2]. Although it can be associated with other congenital defects, only few cases were reported to be associated with an interventricular membranous septal aneurysm leading to obstruction of the right ventricle outflow tract (RVOT). To the best of our knowledge, this is the second reported case in which an aneurysm of the membranous septum dynamically obstructed the RVOT in a child.

## Case presentation

Initially, the boy was evaluated for dyspnoea that had been present since birth. He was diagnosed with a ventricular septum defect (VSD). He was followed by a pediatric cardiologist. He did not receive any medical treatment until the previous year. At the age of four, the transthoracic echocardiography showed a large aneurysm extending to the right ventricular outflow tract and causing RVOT stenosis (peak gradient = 83 mmHg).

The patient was admitted to the cardiac surgery department by the age of five. His mother reported a recurrent upper respiratory tract infection (RTI) throughout his life. The psychomotor assessment reported a delay in speech development, excessive anxiety, and dyspraxia which is also known as developmental co-ordination disorder (DCD). On physical examination, a skull deformity was noted as well as dental abnormalities.

**Fig. 1:**
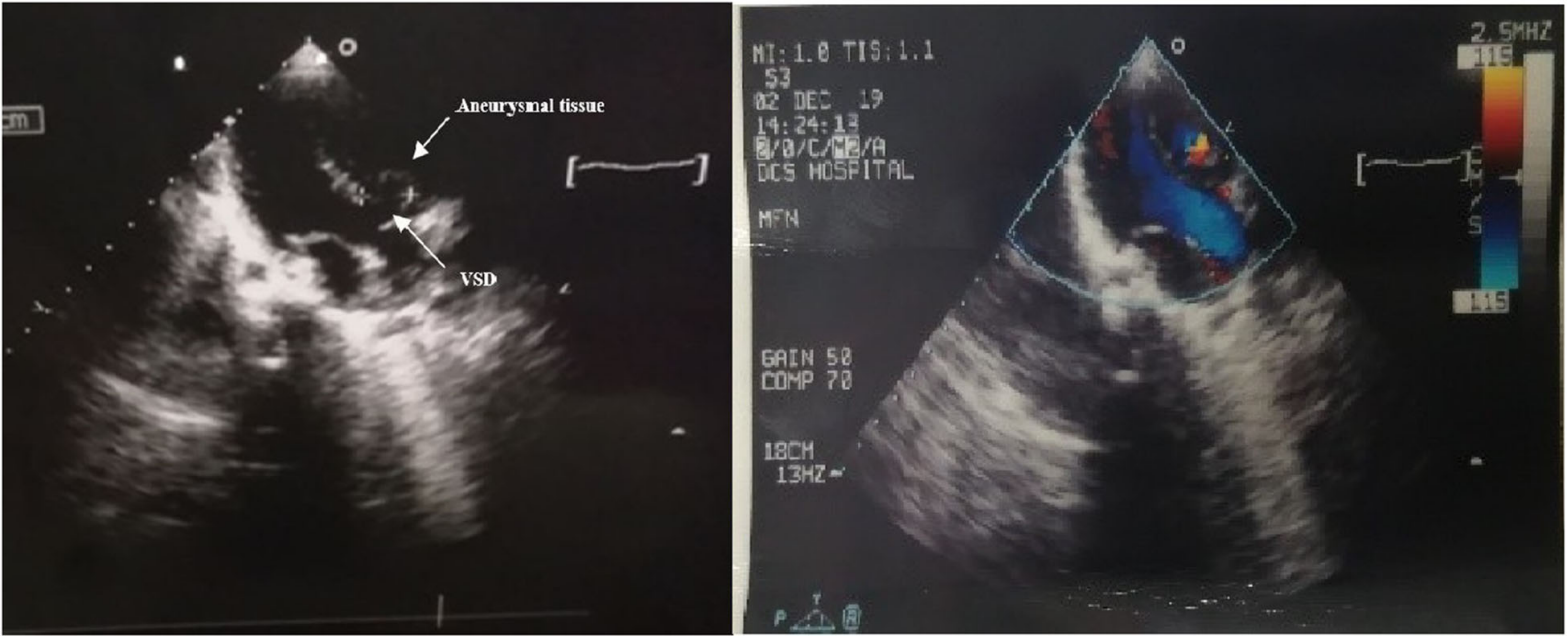
Ventricular septal defect (VSD) along with a large aneurysm of the membranous septum (AVS)

**Fig. 2:**
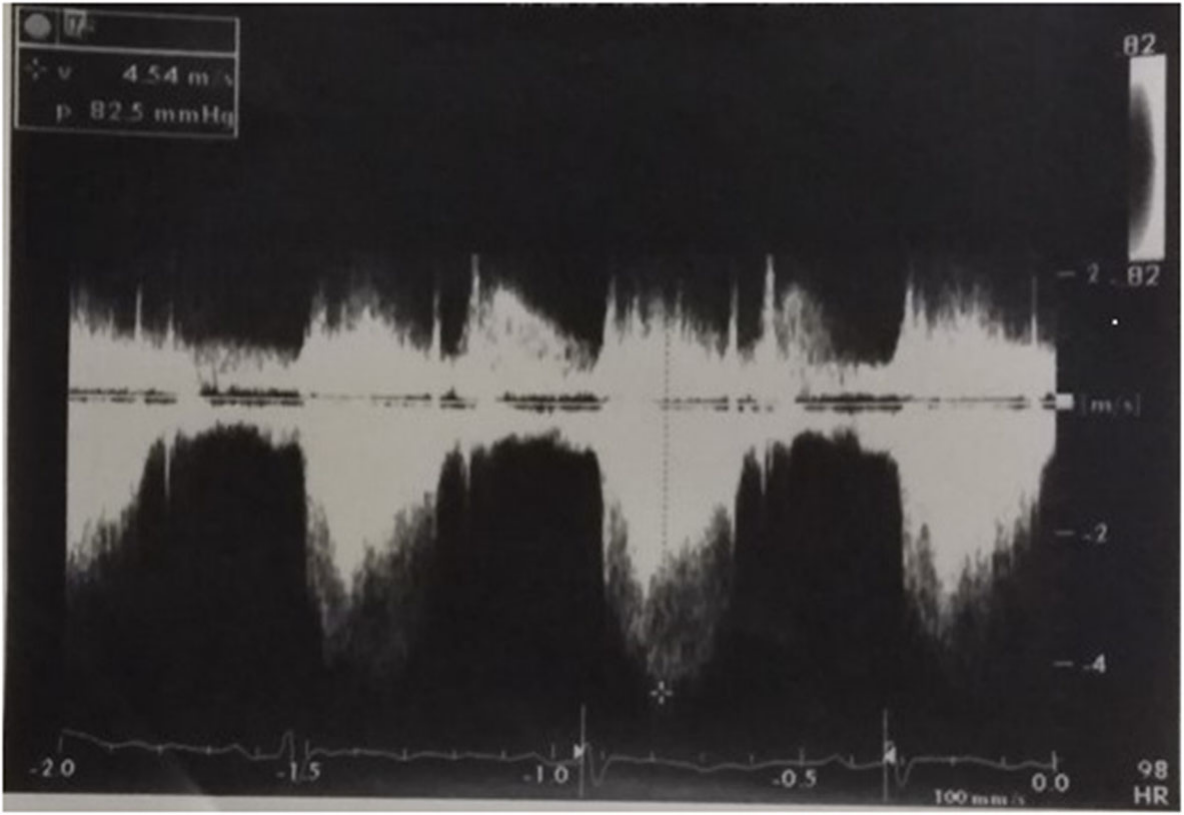
Peak RVOT gradient of 82.5 mmHg in the previous year

**Fig. 3:**
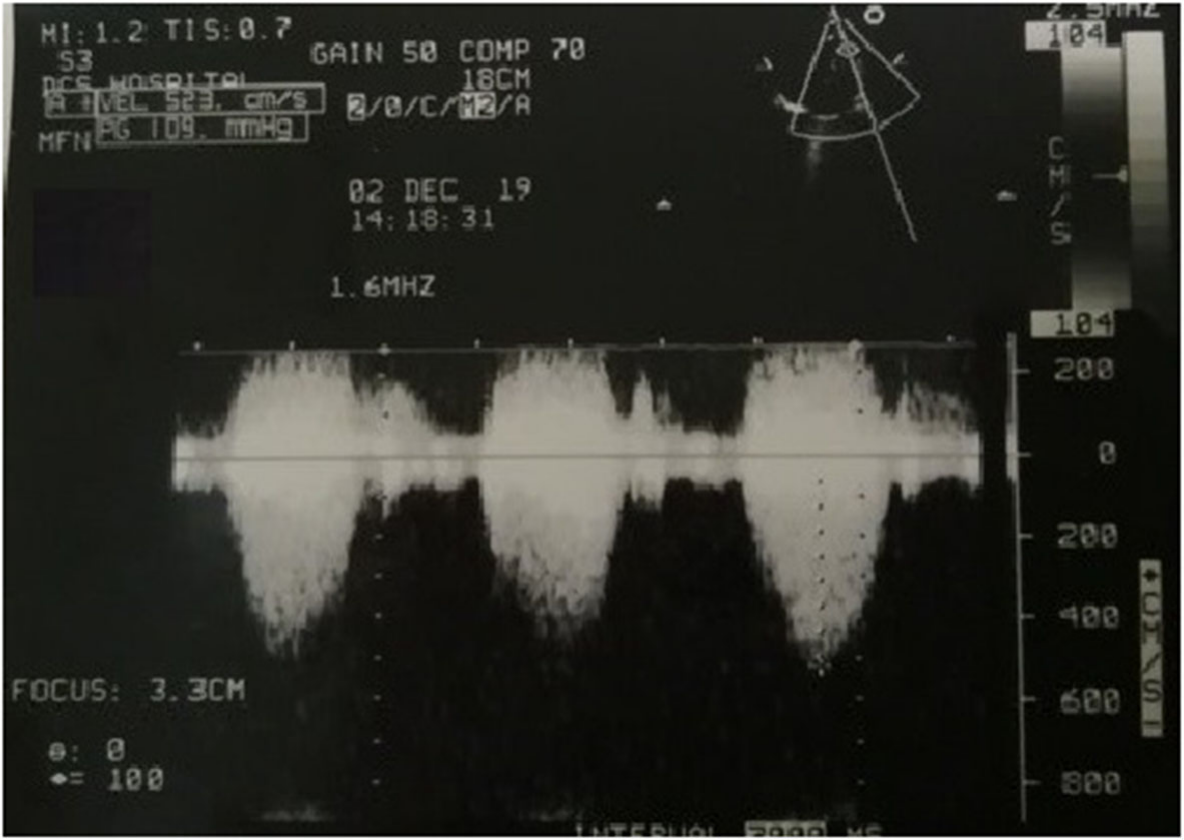
Peak RVOT gradient which elevated to 109 mmHg a few days before the surgery

The preoperative echocardiography (Fig. 1) showed a large VSD (14 × 12 mm) causing a left-to-right shunt, along with an AVS, in addition to a small muscular VSD. The pressure in both ventricles was equal. It also revealed that the peak RVOT gradient elevated from 82.5 mmHg in the previous year (Fig. 2) to 109 mmHg (Fig. 3), which indicated severe RVOT stenosis.

**Fig. 4:**
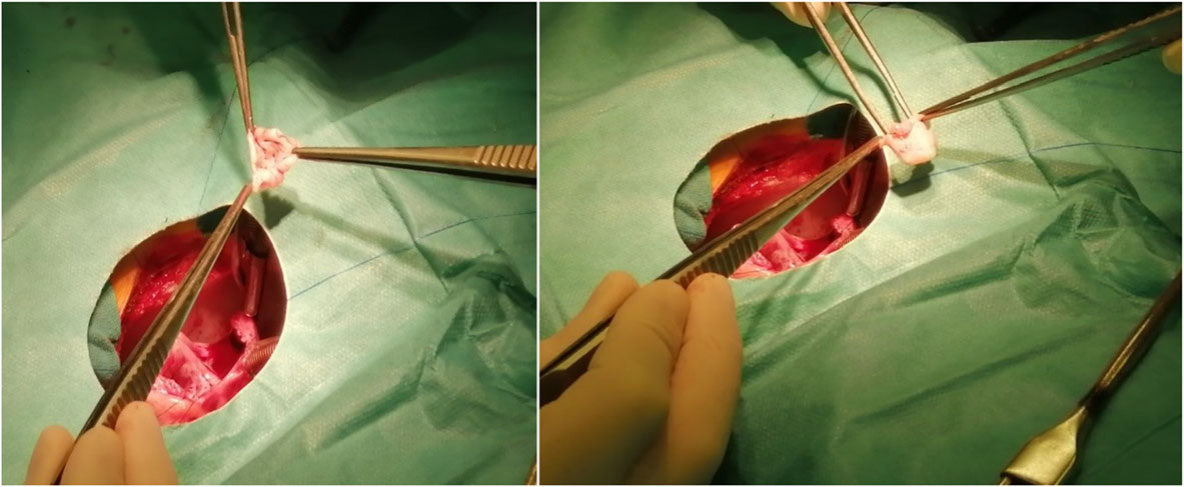
The aneurysmal tissue after complete surgical resection was performed

The surgical procedure was performed through a median sternotomy. Cardiopulmonary bypass was initiated. After the heart was arrested, a right atriotomy was carried out. The RVOT was examined through the tricuspid valve. The aneurysm was found to be a large pouch of membranous tissue, measuring 15 × 20 mm, originating from the superior part of the membranous ventricular septum beneath the aortic valve cusps. During systole, this aneurysm was bulging into the RVOT and causing a severe obstruction of the RVOT. The pulmonary valve had normal leaflets, and no muscular infundibular obstruction was found. The aneurysm was completely resected (Fig. 4). A Dacron patch was finally sewn into place to close the VSD using the interrupted suture technique. The RVOT gradient was calculated as the difference between simultaneously transduced RV and pulmonary artery (PA) systolic pressures, and found to be 10 mmHg following the closure of the VSD.

The patient’s postoperative course was uncomplicated. The postoperative transthoracic echocardiography was performed 5 days after surgery. It revealed a peak RVOT gradient of 17 mmHg, and the VSD was successfully closed. He was discharged home in satisfactory condition.

## Discussion and conclusions

The aneurysm of the membranous septum (AVS) is a congenital lesion that is usually associated with other congenital defects [3]. Aneurysm formation in the membranous septum is a common mechanism to reduce interventricular shunting in patients with ventricular septal defects and is believed to be usually benign in nature [4], and only an occasional finding [2]. This was an unusual case because the AVS caused such a severe obstruction of the RVOT.

An aneurysm of the membranous ventricular septum is a tissue dilatation of this portion, and since it is weak, it bulges to the right ventricle (RV) as a consequence of left ventricle pressure [2]. The most widely accepted theory regarding the etiology of a membranous septum aneurysm is that it forms during the course of spontaneous closure or diminution in the size of a ventricular septal defect (VSD) [3].

We could not find any previous data on the aneurysm of the membranous septum (AVS) in Syria. It occurs in 0.3% of patients with congenital heart disease [2]. However, one of the reported cases was in a 71-year-old woman with a history of previous myocardial infarction [5]. If aneurysms are rare (0.3% of congenital heart disease), the possibility that they cause obstruction is even rarer [2]. To the best of our knowledge, 11 similar cases have been reported since 1982. Before that, three articles mentioned it [6–8]. Only two of those 11 cases [4, 9] were in young children. One of these was in a child with dextrocardia and L-TGA [9] known as the so-called ‘windsock syndrome’ [10], which makes our case the second reported case in children in which an aneurysm of the membranous septum dynamically obstructed the RVOT. What makes this case more unique is that no muscular infundibular obstruction was found, although hypertrophied muscle was mentioned to be resected on the anterior wall of the right ventricle in the first reported case [4]. This indicates that the aneurysm was large enough to cause obstruction without any associated hypertrophied muscle.

Although other cases were diagnosed by transesophageal echocardiography [1] and confirmed by angiography, this was not necessary in our case.

The dyspnoea in this case was an ordinary finding among other cases [2, 11, 12]. However, one of the reported cases presented with chest pain and ventricular tachycardia [5], but that was the case with previous myocardial infarction. The skull and dental deformities should have warranted a genetics evaluation [13]. However, it could not be carried out due to the difficult circumstances. We could not review some of the previously reported cases thoroughly, as the full article was not available online [1, 14].

In conclusion, the occurrence of RVOT obstruction by a membranous ventricular septal aneurysm is very rare. Further studies are needed to obtain a more comprehensive understanding of aneurysms of the membranous septum (AVS).

## Data Availability

The authors declare that the data supporting the findings of this study are available within the article and its supplementary information files.

## Abbreviations

AVS: Aneurysm of the membranous septum
DCD: Developmental co-ordination disorder
PA: Pulmonary artery
RTI: Recurrent upper respiratory tract infection
RV: Right ventricle
RVOT: Right ventricle outflow tract
VSD: Ventricular septal defect
